# The Cascade of Care for an Australian Community-Based Hepatitis C Treatment Service

**DOI:** 10.1371/journal.pone.0142770

**Published:** 2015-11-12

**Authors:** Amanda J. Wade, Diana M. Macdonald, Joseph S. Doyle, Adam Gordon, Stuart K. Roberts, Alexander J. Thompson, Margaret E. Hellard

**Affiliations:** 1 Centre for Population Health, Burnet Institute, Melbourne, Victoria, Australia; 2 School of Public Health and Preventive Medicine, Monash University, Alfred Hospital, Melbourne, Victoria, Australia; 3 Department of Infectious Diseases, The Alfred, Melbourne, Victoria, Australia; 4 Department of Gastroenterology, St Vincent’s Hospital, Melbourne, Victoria, Australia; 5 Department of Gastroenterology, The Alfred, Melbourne, Victoria, Australia; 6 Department of Medicine, Monash University, Melbourne, Victoria, Australia; 7 Department of Medicine, The University of Melbourne, Melbourne, Victoria, Australia; University of Montreal Hospital Research Center (CRCHUM), CANADA

## Abstract

**Background:**

Hepatitis C treatment uptake in Australia is low. To increase access to hepatitis C virus treatment for people who inject drugs, we developed a community-based, nurse-led service that linked a viral hepatitis service in a tertiary hospital to primary care clinics, and resulted in hepatitis C treatment provision in the community.

**Methods:**

A retrospective cohort study of patients referred to the community hepatitis service was undertaken to determine the cascade of care. Logistic regression analyses were used to identify predictors of hepatitis C treatment uptake.

**Results:**

Four hundred and sixty-two patients were referred to the community hepatitis service; 344 attended. Among the 279 attendees with confirmed chronic hepatitis C, 257 (99%) reported ever injecting drugs, and 124 (48%) injected in the last month. Of 201 (72%) patients who had their fibrosis staged, 63 (31%) had F3-F4 fibrosis. Fifty-five patients commenced hepatitis C treatment; 26 (47%) were current injectors and 25 (45%) had F3-F4 fibrosis. Nineteen of the 27 (70%) genotype 1 patients and 14 of the 26 (54%) genotype 3 patients eligible for assessment achieved a sustained virologic response. Advanced fibrosis was a significant predictor of treatment uptake in adjusted analysis (AOR 2.56, CI 1.30–5.00, p = 0.006).

**Conclusions:**

Our community hepatitis service produced relatively high rates of fibrosis assessment, hepatitis C treatment uptake and cure, among people who inject drugs. These findings highlight the potential benefits of providing community-based hepatitis C care to people who inject drugs in Australia–benefits that should be realised as direct-acting antiviral agents become available.

## Introduction

Around 230,000 people are infected with hepatitis C in Australia but annually fewer than 2% are treated and cured [1, 2]. Globally treatment uptake rates are similarly low [3]. People infected with hepatitis C virus (HCV) who do not receive treatment are at risk of developing cirrhosis, liver failure and hepatocellular carcinoma [3]. The highest prevalence of hepatitis C virus (HCV) infection is in people who inject drugs (PWID) or who have a history of injecting drug use, and treatment uptake is particularly low in this group [4, 5]. The reasons for poor treatment uptake are multifactorial and include long and often toxic treatment regimens, difficulties in accessing care provided from tertiary hospitals by specialists, stigma from health service providers and historically, policies of excluding current PWID from treatment [6–8].

Fortunately the HCV treatment landscape is changing; with the development of highly effective, safe and well-tolerated direct-acting antiviral (DAA) therapy many treatment associated barriers have been overcome. Also some policy based barriers have been challenged, with recent clinical guidelines highlighting that injecting drug use is no longer a contraindication to hepatitis C treatment and that HCV treatment should not be withheld based on risk of reinfection [9–11]. These changes offer great hope for the future, however for DAA therapy to have maximal impact on HCV prevalence, morbidity and mortality, health service barriers to HCV treatment also need to be addressed.

Victoria is the second most populous state in Australia with an estimated 55,000 people living with hepatitis C many of whom became infected due to injecting drug use [12]. The capital of Victoria is Melbourne, a city in which the prevalence of HCV in PWID is estimated to be 50%, and treatment rates annually in this group are estimated to be three per 1000 [13]. Recent modeling data has suggested that increasing treatment uptake to 40 per 1000 annually with DAA therapy could halve the prevalence of hepatitis C in 15 years [13]. For this to be achieved a significant change in service delivery would be required.

Treatment for hepatitis C in Australia is provided by specialist services in tertiary hospitals. To be involved with HCV treatment general practitioners must complete a government mandated prescriber course. Post completion of the course a general practitioner cannot initiate HCV therapy, but can prescribe maintenance therapy under the guidance of a specialist physician. In order to increase access to care, in 2011 the Victorian Department of Health funded a new community based, nurse led service which linked primary care clinics to large, tertiary-level HCV treatment centres. Ten community hepatitis nurses were employed to offer hepatitis C assessment and treatment support to PWID, in the community. A number of studies have demonstrated that engaging PWID in community based hepatitis C treatment can be successful [14–16]. In this article, we describe the cascade of care for patients referred to a community hepatitis nurse and the predictors of treatment uptake. This data could be used to inform health service policy, as increasing access to DAA treatment for PWID will decrease the transmission, morbidity and mortality of hepatitis C in our community.

## Methods

### Patients / clinical model

The Alfred Hospital is a tertiary-level university-affiliated teaching hospital in Melbourne, Australia. Since April 2011 the Alfred community hepatitis nurse has worked in three outreach clinics, two located in nearby inner-city suburbs and one regional center 60km (approx. 37 miles) from Melbourne. These clinics provide multidisciplinary health services to PWID, including allied health, primary care and opioid substitution therapy (OST).

Patients are referred to the community hepatitis nurse because of risk behavior or a positive hepatitis C antibody or a positive HCV RNA. The community hepatitis nurse triages referrals from allied health staff and general practitioners working in the outreach clinics. Patient-initiated referrals are also accepted. The initial nursing appointment includes collection of a standardized clinical history, harm reduction education, referral to other services such as mental health or housing if required, and ordering work-up blood tests including HIV serology and transient elastography (TE) (FibroScan, Echosens). TE services are located at the Alfred Hospital. At follow-up appointments, the nurse discusses results and management options with the patient. Hepatitis B vaccination is provided. If the patient is interested in pursuing treatment, an appointment is made for them to attend a specialist physician (in infectious diseases or gastroenterology) for further management. The specialist physicians are located at the outreach clinic for the two inner-city clinics, and at a nearby hospital for the regional clinic.

Most patients who commence treatment receive support and monitoring from the community hepatitis nurse in the community, but selected patients are reviewed at the Alfred Hospital (for example, those commencing first-generation protease-inhibitor-based therapy or requiring specialist cirrhosis care). Also, patients electing to participate in clinical trials of DAA therapy are required to attend the Alfred Hospital for medical appointments.

The standardized clinical history records the following information: demographics including Aboriginal ethnicity, country of origin and language spoken at home; clinical and diagnostic information about hepatitis C; and drug, alcohol and psychiatric history, and is scanned into the electronic medical record.

### Data collection

All patients identified as having been referred to the community hepatitis service (from electronic and paper scheduling data) from April 2011 until August 2014 were retrospectively reviewed. Digital medical records were examined for each referral.

The following data were collected and entered into a Microsoft Access database: demographics; number of appointments scheduled and attended with the community hepatitis nurse and specialist physicians; investigations requested and results; drug, alcohol and psychiatric history on engaging with the service and on starting treatment; treatment episodes and outcome. Current PWID was defined as injecting within the last six months. A fibrosis score of F3-F4 was defined as a liver stiffness score >9.0 kPa on FibroScan, or endoscopic evidence of oesophageal varices. All patients at 12 or more weeks post-treatment completion or cessation were eligible for sustained virologic response (SVR) assessment.

### Clinical outcomes / endpoints

The combined primary endpoint of the study was:

1. attendance at the community hepatitis clinic, defined as attending an appointment with the community hepatitis nurse, and
2. completion of diagnostic blood tests.

For patients diagnosed with chronic hepatitis C (HCV antibody and RNA positive) the primary endpoints were:

1. completion of a FibroScan and HCV genotype,
2. attendance at specialist physician appointment,
3. commencement of hepatitis C therapy defined as self-administration of tablets or injections, and
4. SVR, defined as a negative hepatitis C RNA PCR at 12 or 24 weeks after cessation of therapy; patients who did not have blood collected for PCR assessment 6 or 24 weeks post treatment, were considered lost to follow-up.

The term ‘cascade of care’ refers to the proportion of patients that fulfilled each of the primary endpoints listed above. As data regarding treatment recommendation or treatment deferral was not recorded uniformly throughout patient records, it was not possible to collect retrospectively.

The secondary endpoint was retention in care, defined as the proportion of patients with chronic hepatitis C who first attended the service before August 2013 who had attended the service again between August 2013 and August 2014.

### HCV testing methods

The HCV COBAS AMPLICOR test was used to determine HCV RNA qualitative results. HCV genotyping was done using the Bayer line probe assay, LiPA 11 or Abbott RealTime HCV PCR. HCV quantification was performed using the Bayer HCV 3.0 bDNA or COBAS Taqman HCV test v2.0 test.

### Antiviral therapy availability

PEG and ribavirin dual therapy has been widely available in Australia since June 2003. First-generation protease inhibitors boceprevir and telaprevir were listed on Australia’s Pharmaceutical Benefits Scheme (PBS) in July 2012, and prescribed according to PBS protocol [17]. Access to interferon-free DAA therapy is currently via clinical trials only.

### Statistical analysis

Descriptive statistics were used to report the baseline patient characteristics. Logistic regression analyses were used to identify predictors of HCV treatment uptake. Potential predictors of treatment uptake were determined from the literature and included age (< versus ≥ 35 years), sex, ethnicity (overseas born v non-overseas born), use of psychotropic medication (in last month), previous injecting drug use (in last month), use of OST (in last month), hazardous alcohol use (< or ≥ 4 standard drinks daily), and level of fibrosis (< or ≥F3 fibrosis stage) [18–23]. There were too few co-infections with HIV (n = 1) or hepatitis B virus (HBV) (n = 3) for meaningful comparisons on treatment uptake to be made so these factors were not included in the regression modeling. The multivariate model for predictors of treatment uptake included age, sex and explored factors that were significant at the 0.10 level in univariate analysis in a backwards stepwise model. Statistically significant differences were assessed at the 0.05 level. Data were analyzed using Stata 13.1.

### Ethics approval

The study was approved by the Alfred Hospital and Monash University Ethics Committees. Written informed consent was not required by participants for the use of their clinical records in this study, as patient information was de-identified prior to analysis.

## Results

### Cascade of care

**Fig 1:**
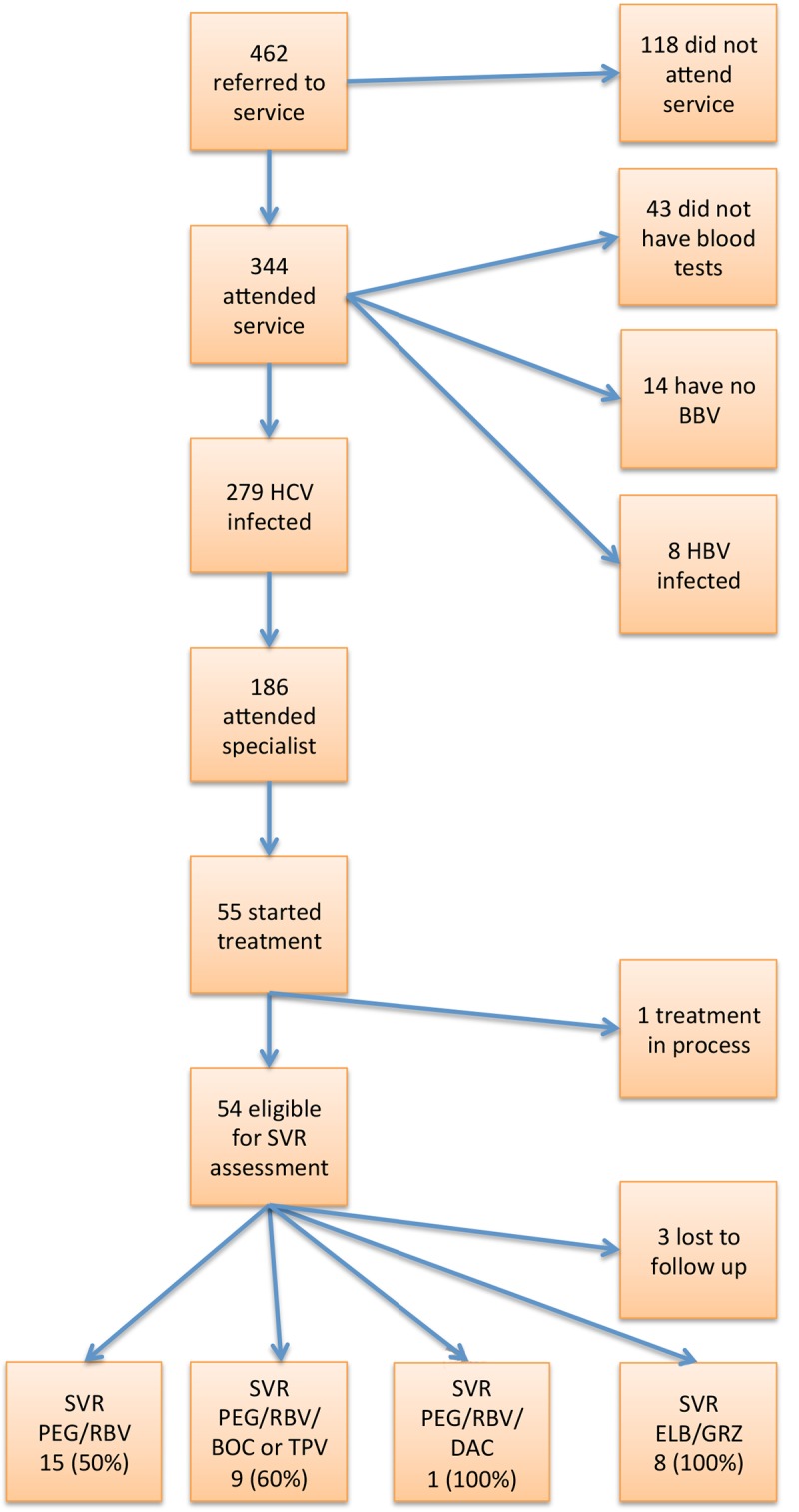
Patient recruitment.

Four hundred and sixty-two patients were referred to the community hepatitis nurse in the 3.4 year study period. Patient recruitment is shown in Fig 1. Of the 344 who attended the service, 279 were diagnosed with chronic HCV infection. Two hundred and seventy five patients had HCV mono-infection (HCV antibody positive and HCV RNA positive), three had HBV-HCV co-infection and only one patient had HIV-HCV co-infection. Eight patients had HBV mono-infection (HBsAg positive). Fourteen patients screened negative for all three blood-borne viruses. Forty-three patients did not have blood tests and declined further follow-up with the service.

### Patient characteristics

Two hundred and seventy-nine patients aged 19–64 years with chronic HCV were included in the analysis. Table 1 shows the patients’ demographic and clinical characteristics. Nearly all patients had ever injected drugs (99%), half (48%) reported injecting in the past month and most (61%) were on OST. Most (85%) patients had a psychiatric diagnosis and (65%) were on psychotropic medication. Of the 201 (72%) patients who had their fibrosis staged, 199 had a valid FibroScan and 61 of these patients had a liver stiffness score >9.0 kPa indicating F3-F4 fibrosis. Another two patients had endoscopic evidence of oesophageal varices, therefore 63 (31%) of the patients who had fibrosis assessment had F3-F4 fibrosis. Compared to the patients with F0-F2 fibrosis, the patients with F3-F4 were older (46 years vs 42 years), had higher baseline ALT (155 U/L vs 72 U/L) and higher alcohol intake (35% vs 20% consumed more than 2 standard drinks per day in the month before engaging with the service).

**Table 1:** Patient demographic and clinical characteristics.

*Variable (Number of available data)*	*N (N = 279)*	*Proportion or Mean (SD) or Median (range)*
*Gender (n = 279)*		
*Male*	*184*	*66%*
*Age (n = 274)*		*mean 42 (9*.*1)*
*< 35 years*	*63*	*23%*
*> 35 years*	*211*	*77%*
*Overseas born or English not spoken at home (n = 210)*	*16*	*8%*
*Aboriginal ethnicity (n = 209)*	*10*	*5%*
*Psychiatric diagnosis (n = 246)*	*208*	*85%*
*On psychotropic medication† (n = 223)*	*145*	*65%*
*Ever injected drugs (n = 259)*	*257*	*99%*
*OST in month prior to engagement† (n = 240)*	*147*	*61%*
*Injected drugs in month prior to engagement† (n = 257)*	*124*	*48%*
*Frequency of injecting per month† (n = 119)*		
*1–2*	*56*	*47%*
*3–4*	*17*	*14%*
*5–10*	*17*	*14%*
*11–20*	*12*	*10%*
*>28*	*17*	*14%*
*Alcohol intake average no*. *std drinks/day in prior month† (n = 236)*		
*0*	*97*	*41%*
*<2*	*76*	*32%*
*2–4*	*16*	*7%*
*4–10*	*34*	*14%*
*>10*	*13*	*6%*
*HCV genotype (n = 267)*		
*Genotype 1*	*128*	*46%*
*Genotype 3*	*127*	*46%*
*Genotype other*	*12*	*4%*
*HCV viral load (IU/ml) (n = 105)*		
*>400*,*000*	*62*	*59%*
*<400*,*000*	*43*	*41%*
*HIV co-infection (n = 230)*	*1*	*0*.*4%*
*HBV co-infection (n = 243)*	*3*	*1%*
*FibroScan kPa (n = 199)*		*median 6*.*4 (3*.*2–75)*
*Fibrosis assessment (n = 201)*		
*F0-F2*	*138*	*69%*
*F3-4*	*63*	*31%*
*ALT (U/L) (n = 264)*		*mean 89 (114)*
*Albumin (g/L) (n = 261)*		*mean 39 (3*.*9)*
*Platelets (x10* ^*9*^ */L) (n = 256)*		*mean 213 (76)*

† In month prior to engaging with community hepatitis service; OST opiate substitution therapy.

### Attendance, treatment uptake and retention in care

Of the 279 patients reviewed by the community hepatitis nurse and identified as having hepatitis C infection, 186 (67%) attended a specialist physician appointment.

**Fig 2:**
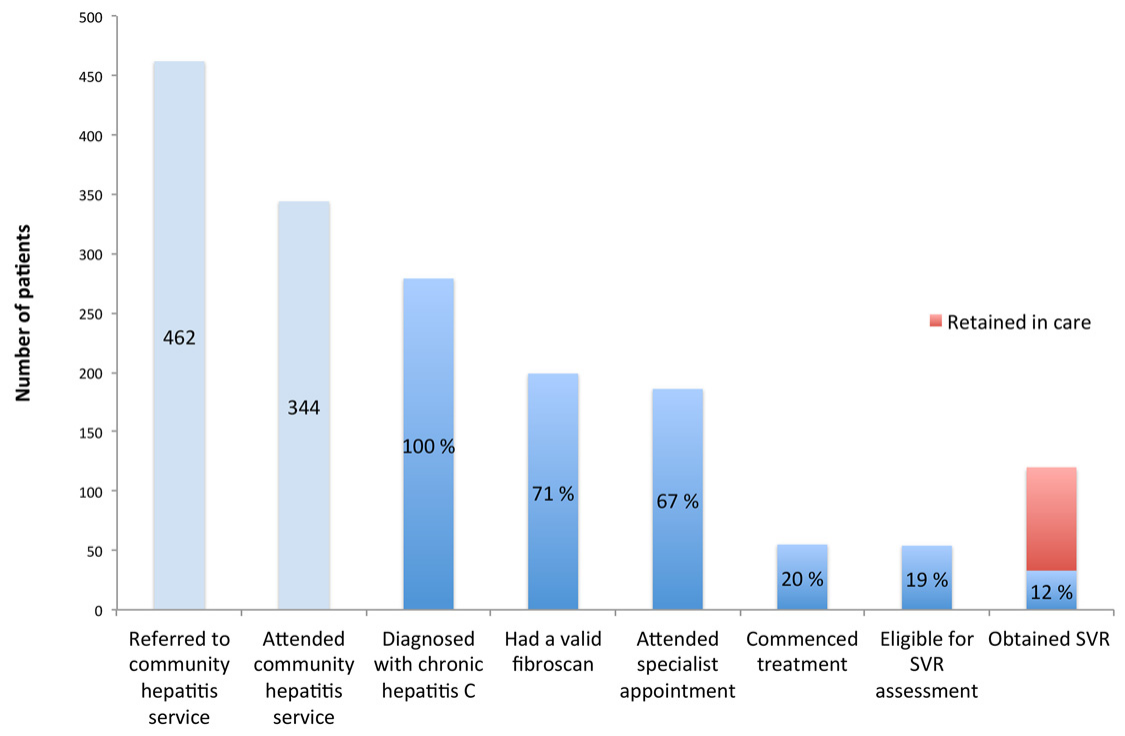
Cascade of care.

Fifty-five patients (20%) commenced HCV therapy during the study period (Fig 2 Cascade of care). Of these patients, 25 (45%) had F3-F4 fibrosis. The Il28B status of patients who commenced therapy was CC 14 (26%), CT 20 (36%), TT 3 (5%), test not performed 18 (33%). Of the 55 patients who commenced therapy; 26 (47%) were current injectors, 25 (45%) were not current injectors, and data were missing for four patients.

Twenty-eight patients with HCV genotype 1 commenced therapy; of these 11 (39%) had F3-F4 fibrosis. Three patients were treated with PEG plus ribavirin therapy, of whom one achieved SVR. Fifteen patients were treated with protease-inhibitor-based therapy with telaprevir or boceprevir combined with PEG plus ribavirin, with nine achieving an SVR. Of the remaining six patients, four fulfilled futility criteria, and two were lost to follow up. One patient was successfully treated with PEG, ribavirin and daclatasvir via a clinical trial. In another clinical trial eight patients achieved an SVR with grazoprevir (GRZ) and elbasvir (ELB) whilst one additional patient is not yet 12 weeks post treatment completion. Overall, 19 of the 27 (70%) genotype 1 patients eligible for SVR assessment achieved an SVR, six did not and two were lost to follow-up.

Twenty-six patients with HCV genotype 3 commenced treatment with PEG and ribavirin therapy, 14 (54%) of whom had F3-F4 fibrosis. Sixteen completed the treatment course, nine did not complete therapy, and it is unclear if one patient completed therapy due to incomplete medical records. Of the patients who did not complete therapy, three had psychiatric complications requiring treatment cessation, four were non-compliant, one fulfilled futility criteria, and one had hypoxia and pulmonary interstitial markings as a possible drug reaction, necessitating treatment cessation. Fourteen of the 26 (54%) genotype 3 patients achieved an SVR, 11 did not, and one was lost to follow up.

One patient with HCV genotype 2 commenced treatment with PEG and ribavirin therapy. The patient was unable to finish treatment because of intractable vomiting, and did not obtain an SVR.

Overall, of the 55 patients who commenced therapy, 54 (98%) were eligible for SVR assessment and one (2%) had not yet reached the threshold of 12 weeks post-treatment. Thirty-three of 54 patients eligible for SVR assessment (61%) achieved an SVR, 18 (33%) did not and 3 (6%) were lost to follow-up.

Of the 279 patients who attended the service and had HCV infection, 92 (33%) were referred for formal psychiatric assessment. Forty of 66 patients (61%) who attended a psychiatry appointment commenced treatment, while none of the 18 patients who did not attend commenced HCV treatment. For eight patients, data about psychiatry attendance were not available.

Of 246 patients with chronic HCV who did not undergo treatment, or if treated had not completed therapy or did not have an SVR, 87 (35%) were retained in care.

### Factors associated with treatment uptake

Univariate logistic regression analysis demonstrated a significant association between treatment uptake and F3-F4 fibrosis (OR 2.36, 95%CI 1.24–4.52, p = 0.009). F3-F4 fibrosis remained a significant predictor of treatment uptake in multivariate analysis after adjusting for age and gender (OR 2.56, 95%CI 1.30–5.00, p = 0.006) (see Table 2).

**Table 2:** Unadjusted and adjusted analysis of factors associated with HCV treatment uptake (N = 279).

*Characteristic*	*Untreated (n = 224)*	*Treated (n = 55)*	*OR (95% CI)*	*P value*	*Adjusted OR (95%CI)*	*P value*
*Age*						
*< 35 years*	*51*	*12*	*1*.*00*			
*> 35 years*	*168*	*43*	*1*.*08 (0*.*53–2*.*21)*	*0*.*81*	*0*.*75 (0*.*34–1*.*66)*	*0*.*48*
*Gender*						
*Male*	*144*	*40*	*1*.*00*			
*Female*	*80*	*15*	*0*.*67 (0*.*35–1*.*29)*	*0*.*23*	*0*.*79 (0*.*39–1*.*61)*	*0*.*56*
*Country of origin / language at home*						
*Australian born or English spoken at home*	*165*	*29*	*1*.*00*			
*Overseas born or English not spoken at home*	*12*	*4*	*1*.*89 (0*.*57–6*.*23)*	*0*.*29*		
*Fibrosis stage*						
*F0 –F2*	*108*	*30*	*1*.*00*			
*F3 –F4*	*38*	*25*	*2*.*36 (1*.*24–4*.*52)*	*0*.*009*	*2*.*56 (1*.*30–5*.*00)*	*0*.*006*
*OST†*						
*No*	*73*	*20*	*1*.*00*			
*Yes*	*115*	*32*	*1*.*01 (0*.*54–1*.*90)*	*0*.*96*		
*Injection drug use†*						
*No*	*85*	*21*	*1*.*00*			
*Yes*	*102*	*22*	*0*.*87 (0*.*44–1*.*69)*	*0*.*68*		
*Alcohol consumption†*						
*0–4 std drinks per day*	*154*	*35*	*1*.*00*			
*>4 std drinks per day*	*39*	*8*	*0*.*90 (0*.*39–2*.*1)*	*0*.*81*		
*Psychotropic medication†*						
*No*	*63*	*15*	*1*.*00*			
*Yes*	*110*	*35*	*1*.*33 (0*.*67–2*.*63)*	*0*.*40*		

† In month prior to engaging with service.

## Discussion

This retrospective cohort study of PWID referred to a community hepatitis service highlights multiple opportunities for improvement along the hepatitis C cascade of care. Outcomes of treatment were acceptable given the high prevalence of F3-F4 fibrosis in the treatment group, with 70% of patients with genotype 1 and 54% with genotype 3 achieving an SVR.

Seventy two percent of the patients infected with HCV who attended the community hepatitis service underwent TE, with over 30% having evidence of F3-F4 fibrosis. Our finding that treatment uptake was significantly associated with F3-F4 fibrosis is consistent with other studies, and demonstrates the importance of TE accessibility [8, 19]. Interferon free DAA regimens were available only through clinical trials during the study period, and therefore deferral of treatment was commonly advised to patients with low levels of fibrosis. Clinicians were probably more likely to advise patients with F3 and F4 fibrosis to expedite treatment, and patients more motivated to seek out treatment. Interestingly 53% of the patients that received pegylated interferon and ribavirin therapy had F3 F4 fibrosis, compared to 40% of the patients that received pegylated interferon, ribavirin and a first generation protease inhibitor, compared to 33% of the patients that received interferon free therapy. The relationship between advanced fibrosis and treatment uptake is likely to vary according to treatment regimen; when all oral regimes become available it is likely there will be an increase in treatment uptake in patients with less advanced fibrosis scores.

Studies in conventional tertiary settings have reported treatment uptake in PWID of 1–6% [5, 23]. Iversen et al investigated HCV treatment uptake rates in people with positive HCV antibody attending the needle and syringe exchange program in Australia, and demonstrated in 2011 8.6% of this group had ever received treatment for HCV [21]. During the 3.4 year study period, our service treated 55 patients and had a treatment uptake rate of 20%. This is concordant with previous cohort studies of hepatitis services specifically established to improve treatment uptake in OST centers and resulted in treatment uptake rates of 19–22% [14, 19].

A small number of published studies compare treatment uptake by service delivery. Mousalli et al noted an increase in treatment uptake when provided at an OST clinic. Before treatment was available in the OST clinic two of 337 patients commenced treatment for HCV. After treatment was made available in the OST clinic 85 patients (25%) commenced therapy [24]. Gigi et al demonstrated higher rates of treatment uptake in an OST clinic compared to a tertiary hospital clinic (61% vs 55%) [25]. To our knowledge, the only randomised trial regarding the impact of placement of HCV treatment services in OST clinics on treatment uptake was conducted by Bruce et al, and included modified directly observed therapy [16]. Subjects on methadone in an OST clinic were randomized to receive modified directly observed treatment at the OST clinic or standard of care therapy at a tertiary liver clinic. Subjects treated at the OST clinic had directly observed therapy for methadone, pegylated interferon and morning ribavirin doses, but self administered evening ribavirin. All 12 patients randomized to the OST clinic started treatment and six of eight patients (75%) eligible to be assessed for SVR achieved SVR. In comparison four of the nine patients (44%) randomized to standard of care, commenced treatment and one of three (33%) of patients eligible to be assessed for SVR achieved an SVR [16].

Whilst a large randomised trial is required to conclude that provision of HCV treatment in the community increases treatment uptake, our study and evidence in the literature suggests that provision of HCV treatment in primary care clinics that provide OST services will increase HCV treatment uptake.

Studies describing the hepatitis C care cascade from diagnosis to cure demonstrate attrition of the patient population at every step in the pathway [26, 27]. Provision of DAA therapy will eliminate many treatment-based barriers to HCV care, but service-based barriers also need to be addressed. Provision of treatment in the community [14, 27] and access to care via telehealth [28–30] are likely to overcome some of the service-based barriers and increase treatment uptake. Our model of nurse led HCV care would readily adapt to the provision of DAA therapy, allowing nurse practitioners to play a key role in community based HCV treatment, education and harm minimization.

Limitations of this study include its retrospective nature, resulting in varying degrees of missing behavioural data, as listed in Table 1. As per standard practice all patients with HCV infection were offered Fibroscan, but acceptance is patient driven, and as such only 72% completed the test. However similar rates of F3 F4 fibrosis were observed in a cohort of community patients, in which 24% had a FibroScan result greater than 9.5kpa (personal communication, S Roberts). Patients who did not attend the community hepatitis service may have sought care and treatment elsewhere.

In conclusion, this community hepatitis service successfully provided care and treatment to several dozen PWID with HCV infection, with an SVR rate comparable to patients treated in tertiary institutions. Highly potent DAAs have made eradicating hepatitis C conceivable, but for this to occur delivery systems must address the complex and wide-ranging difficulties associated with HCV in the community. This study highlights the potential of community-based HCV services to manage PWID, a group with a high prevalence of HCV infection.

## References

[1] Gidding HF, Topp L, Middleton M, Robinson K, Hellard M, McCaughan G, et al The epidemiology of hepatitis C in Australia: notifications, treatment uptake and liver transplantations, 1997–2006. Journal of gastroenterology and hepatology. 2009;24(10):1648–54. Epub 2009/10/03. . 19798783 10.1111/j.1440-1746.2009.05910.x

[2] Australias notifiable disease status 2011:Annual report of the National Notifiable Diseases Surveillance System. In: Health, editor. http://www.health.gov.au/internet/main/publishing.nsf/Content/cda-surveil-nndss-2011-annual-report.htm2011. 10.33321/cdi.2013.37.49 24882235

[3] Dore GJ, Ward J, Thursz M. Hepatitis C disease burden and strategies to manage the burden (Guest Editors Mark Thursz, Gregory Dore and John Ward). Journal of viral hepatitis. 2014;21 Suppl 1:1–4. Epub 2014/04/10. 10.1111/jvh.12253 . 24713003

[4] Nelson PK, Mathers BM, Cowie B, Hagan H, Des Jarlais D, Horyniak D, et al Global epidemiology of hepatitis B and hepatitis C in people who inject drugs: results of systematic reviews. Lancet. 2011;378(9791):571–83. Epub 2011/08/02. 10.1016/s0140-6736(11)61097-0 ; PubMed Central PMCID: PMCPmc3285467. 21802134 PMC3285467

[5] Grebely J, Raffa JD, Lai C, Krajden M, Kerr T, Fischer B, et al Low uptake of treatment for hepatitis C virus infection in a large community-based study of inner city residents. Journal of viral hepatitis. 2009;16(5):352–8. Epub 2009/02/20. 10.1111/j.1365-2893.2009.01080.x . 19226330

[6] Sublette VA, Smith SK, George J, McCaffery K, Douglas MW. The Hepatitis C treatment experience: Patients' perceptions of the facilitators of and barriers to uptake, adherence and completion. Psychology & health. 2015;30(8):987–1004. Epub 2015/01/28. 10.1080/08870446.2015.1012195 . 25622699

[7] Myles A, Mugford GJ, Zhao J, Krahn M, Wang PP. Physicians' attitudes and practice toward treating injection drug users with hepatitis C: results from a national specialist survey in Canada. Canadian journal of gastroenterology = Journal canadien de gastroenterologie. 2011;25(3):135–9. Epub 2011/04/19. ; PubMed Central PMCID: PMCPmc3076031. 21499577 10.1155/2011/810108 PMC3076031

[8] Crespo J, Cabezas J, Sacristan B, Olcoz JL, Perez R, De la Vega J, et al Barriers to HCV treatment in the era of triple therapy: a prospective multi-centred study in clinical practice. Liver international: official journal of the International Association for the Study of the Liver. 2015;35(2):401–8. Epub 2014/03/22. 10.1111/liv.12536 . 24650000

[9] Robaeys G, Grebely J, Mauss S, Bruggmann P, Moussalli J, De Gottardi A, et al Recommendations for the management of hepatitis C virus infection among people who inject drugs. Clinical infectious diseases: an official publication of the Infectious Diseases Society of America. 2013;57 Suppl 2:S129–37. Epub 2013/08/02. 10.1093/cid/cit302 . 23884061

[10] Aspinall EJ, Corson S, Doyle JS, Grebely J, Hutchinson SJ, Dore GJ, et al Treatment of hepatitis C virus infection among people who are actively injecting drugs: a systematic review and meta-analysis. Clinical infectious diseases: an official publication of the Infectious Diseases Society of America. 2013;57 Suppl 2:S80–9. Epub 2013/08/02. 10.1093/cid/cit306 . 23884071

[11] Grady BP, Schinkel J, Thomas XV, Dalgard O. Hepatitis C virus reinfection following treatment among people who use drugs. Clinical infectious diseases: an official publication of the Infectious Diseases Society of America. 2013;57 Suppl 2:S105–10. Epub 2013/08/02. 10.1093/cid/cit301 . 23884057

[12] EASL Clinical Practice Guidelines: management of hepatitis C virus infection. Journal of hepatology. 2014;60(2):392–420. Epub 2013/12/18. 10.1016/j.jhep.2013.11.003 . 24331294

[13] Martin NK, Vickerman P, Grebely J, Hellard M, Hutchinson SJ, Lima VD, et al Hepatitis C virus treatment for prevention among people who inject drugs: Modeling treatment scale-up in the age of direct-acting antivirals. Hepatology (Baltimore, Md). 2013;58(5):1598–609. Epub 2013/04/05. 10.1002/hep.26431 ; PubMed Central PMCID: PMCPmc3933734. 23553643 PMC3933734

[14] Alavi M, Grebely J, Micallef M, Dunlop AJ, Balcomb AC, Day CA, et al Assessment and treatment of hepatitis C virus infection among people who inject drugs in the opioid substitution setting: ETHOS study. Clinical infectious diseases: an official publication of the Infectious Diseases Society of America. 2013;57 Suppl 2:S62–9. Epub 2013/08/02. 10.1093/cid/cit305 . 23884068

[15] Jack K, Willott S, Manners J, Varnam MA, Thomson BJ. Clinical trial: a primary-care-based model for the delivery of anti-viral treatment to injecting drug users infected with hepatitis C. Alimentary pharmacology & therapeutics. 2009;29(1):38–45. Epub 2008/10/24. 10.1111/j.1365-2036.2008.03872.x . 18945252

[16] Bruce RD, Eiserman J, Acosta A, Gote C, Lim JK, Altice FL. Developing a modified directly observed therapy intervention for hepatitis C treatment in a methadone maintenance program: implications for program replication. American Journal of Drug & Alcohol Abuse. 2012;38(3):206–12. Language: English. Entry Date: 20120824. Revision Date: 20120824. Publication Type: journal article. 10.3109/00952990.2011.643975 22242700 PMC5573865

[17] Australian Government DoH. The Pharmaceutical Benefits Scheme www.pbs.gov.au2015 [24.08.2015]. Public summary document].

[18] Grebely J, Petoumenos K, Matthews GV, Haber P, Marks P, Lloyd AR, et al Factors associated with uptake of treatment for recent hepatitis C virus infection in a predominantly injecting drug user cohort: The ATAHC Study. Drug and alcohol dependence. 2010;107(2–3):244–9. Epub 2009/11/21. 10.1016/j.drugalcdep.2009.09.015 ; PubMed Central PMCID: PMCPmc2853739. 19926405 PMC2853739

[19] Martinez AD, Dimova R, Marks KM, Beeder AB, Zeremski M, Kreek MJ, et al Integrated internist—addiction medicine—hepatology model for hepatitis C management for individuals on methadone maintenance. Journal of viral hepatitis. 2012;19(1):47–54. Epub 2010/12/07. 10.1111/j.1365-2893.2010.01411.x ; PubMed Central PMCID: PMCPmc3708453. 21129131 PMC3708453

[20] Treloar C, Hull P, Dore GJ, Grebely J. Knowledge and barriers associated with assessment and treatment for hepatitis C virus infection among people who inject drugs. Drug and alcohol review. 2012;31(7):918–24. Epub 2012/05/23. 10.1111/j.1465-3362.2012.00468.x . 22612899

[21] Iversen J, Grebely J, Topp L, Wand H, Dore G, Maher L. Uptake of hepatitis C treatment among people who inject drugs attending Needle and Syringe Programs in Australia, 1999–2011. Journal of viral hepatitis. 2014;21(3):198–207. Epub 2014/01/21. 10.1111/jvh.12129 . 24438681

[22] Strathdee SA, Latka M, Campbell J, O'Driscoll PT, Golub ET, Kapadia F, et al Factors associated with interest in initiating treatment for hepatitis C Virus (HCV) infection among young HCV-infected injection drug users. Clinical infectious diseases: an official publication of the Infectious Diseases Society of America. 2005;40 Suppl 5:S304–12. Epub 2005/03/16. 10.1086/427445 ; PubMed Central PMCID: PMCPmc2196220. 15768339 PMC2196220

[23] Mehta SH, Genberg BL, Astemborski J, Kavasery R, Kirk GD, Vlahov D, et al Limited uptake of hepatitis C treatment among injection drug users. Journal of community health. 2008;33(3):126–33. Epub 2008/01/01. 10.1007/s10900-007-9083-3 ; PubMed Central PMCID: PMCPmc3800027. 18165889 PMC3800027

[24] Moussalli J, Delaquaize H, Boubilley D, Lhomme JP, Merleau Ponty J, Sabot D, et al Factors to improve the management of hepatitis C in drug users: An observational study in an addiction centre. Gastroenterology Research and Practice. 2010;((Moussalli, Poynard) Universite Pierre, Marie Curie Liver Center, Hopital la Pitie Salptrire, 75013 Paris, France). 10.1155/2010/261472 PMC2926583 20811482

[25] Gigi E, Sinakos E, Sykja A, Androulakis G, Tanis C, Stayridou V, et al Epidemiology, clinical data, and treatment of viral hepatitis in a large cohort of intravenous drug users. Journal of addiction medicine. 2013;7(1):52–7. 10.1097/ADM.0b013e318279756f 23340710

[26] Yehia BR, Schranz AJ, Umscheid CA, Lo Re V 3rd. The treatment cascade for chronic hepatitis C virus infection in the United States: a systematic review and meta-analysis. PloS one. 2014;9(7):e101554 Epub 2014/07/06. 10.1371/journal.pone.0101554 ; PubMed Central PMCID: PMCPmc4079454. 24988388 PMC4079454

[27] Hill WD, Butt G, Alvarez M, Krajden M. Capacity enhancement of hepatitis C virus treatment through integrated, community-based care. Canadian journal of gastroenterology = Journal canadien de gastroenterologie. 2008;22(1):27–32. Epub 2008/01/23. ; PubMed Central PMCID: PMCPmc2659116. 18209777 10.1155/2008/369827 PMC2659116

[28] Lloyd AR, Clegg J, Lange J, Stevenson A, Post JJ, Lloyd D, et al Safety and effectiveness of a nurse-led outreach program for assessment and treatment of chronic hepatitis C in the custodial setting. Clinical infectious diseases: an official publication of the Infectious Diseases Society of America. 2013;56(8):1078–84. Epub 2013/01/31. 10.1093/cid/cis1202 . 23362288

[29] Nazareth S, Kontorinis N, Muwanwella N, Hamilton A, Leembruggen N, Cheng WS. Successful treatment of patients with hepatitis C in rural and remote Western Australia via telehealth. Journal of telemedicine and telecare. 2013;19(2):101–6. Epub 2013/03/27. 10.1258/jtt.2012.120612 . 23528788

[30] Arora S, Thornton K, Murata G, Deming P, Kalishman S, Dion D, et al Outcomes of treatment for hepatitis C virus infection by primary care providers. The New England journal of medicine. 2011;364(23):2199–207. Epub 2011/06/03. 10.1056/NEJMoa1009370 ; PubMed Central PMCID: PMCPmc3820419. 21631316 PMC3820419

